# Realizing the value in “non-standard” parts of the qPCR standard curve by integrating fundamentals of quantitative microbiology

**DOI:** 10.3389/fmicb.2023.1048661

**Published:** 2023-03-03

**Authors:** Philip J. Schmidt, Nicole Acosta, Alex H. S. Chik, Patrick M. D’Aoust, Robert Delatolla, Hadi A. Dhiyebi, Melissa B. Glier, Casey R. J. Hubert, Jennifer Kopetzky, Chand S. Mangat, Xiao-Li Pang, Shelley W. Peterson, Natalie Prystajecky, Yuanyuan Qiu, Mark R. Servos, Monica B. Emelko

**Affiliations:** ^1^Department of Civil and Environmental Engineering, University of Waterloo, Waterloo, ON, Canada; ^2^Department of Microbiology, Immunology and Infectious Diseases, University of Calgary, Calgary, AB, Canada; ^3^Ontario Clean Water Agency, Mississauga, ON, Canada; ^4^Department of Civil Engineering, University of Ottawa, Ottawa, ON, Canada; ^5^Department of Biology, University of Waterloo, Waterloo, ON, Canada; ^6^Public Health Laboratory, BC Centre for Disease Control, Vancouver, BC, Canada; ^7^Department of Biological Sciences, University of Calgary, Calgary, AB, Canada; ^8^Department of Pathology and Laboratory Medicine, University of British Columbia, Vancouver, BC, Canada; ^9^Wastewater Surveillance Unit, National Microbiology Laboratory, Public Health Agency of Canada, Winnipeg, MB, Canada; ^10^Department of Laboratory Medicine and Pathology, University of Alberta, Edmonton, AB, Canada; ^11^Alberta Precision Laboratories, Public Health Laboratory, Alberta Health Services, Edmonton, AB, Canada; ^12^Li Ka Shing Institute of Virology, University of Alberta, Edmonton, AB, Canada

**Keywords:** quantification cycle, threshold cycle, amplification efficiency, PCR efficiency, non-detects, uncertainty

## Abstract

The real-time polymerase chain reaction (PCR), commonly known as quantitative PCR (qPCR), is increasingly common in environmental microbiology applications. During the COVID-19 pandemic, qPCR combined with reverse transcription (RT-qPCR) has been used to detect and quantify SARS-CoV-2 in clinical diagnoses and wastewater monitoring of local trends. Estimation of concentrations using qPCR often features a log-linear standard curve model calibrating quantification cycle (*Cq*) values obtained from underlying fluorescence measurements to standard concentrations. This process works well at high concentrations within a linear dynamic range but has diminishing reliability at low concentrations because it cannot explain “non-standard” data such as *Cq* values reflecting increasing variability at low concentrations or non-detects that do not yield *Cq* values at all. Here, fundamental probabilistic modeling concepts from classical quantitative microbiology were integrated into standard curve modeling approaches by reflecting well-understood mechanisms for random error in microbial data. This work showed that data diverging from the log-linear regression model at low concentrations as well as non-detects can be seamlessly integrated into enhanced standard curve analysis. The newly developed model provides improved representation of standard curve data at low concentrations while converging asymptotically upon conventional log-linear regression at high concentrations and adding no fitting parameters. Such modeling facilitates exploration of the effects of various random error mechanisms in experiments generating standard curve data, enables quantification of uncertainty in standard curve parameters, and is an important step toward quantifying uncertainty in qPCR-based concentration estimates. Improving understanding of the random error in qPCR data and standard curve modeling is especially important when low concentrations are of particular interest and inappropriate analysis can unduly affect interpretation, conclusions regarding lab performance, reported concentration estimates, and associated decision-making.

## 1. Introduction

The standard curve is a mathematical cornerstone for estimating concentrations of target genes from the fluorescence data measured in real-time polymerase chain reactions. It is essentially an empirical linear calibration between log-concentration of target genes and the quantification cycle (*Cq*) at which measured fluorescence reaches some threshold after adjusting for background fluorescence (Rutledge and Côté, 2003; Bustin et al., 2009). Under idealized but sometimes practically relevant conditions (i.e., high concentrations, accurately quantified standards, precisely controlled dilution, low variation in *Cq* values among technical replicates), simply mapping *Cq* values to point-estimates of concentration using a standard curve can be accurate. Numerous alternative methods have also been developed to extract information from raw fluorescence curves of individual reactions (Ruijter et al., 2013), often with the explicit goal of eliminating the need for standard curves (Rutledge and Stewart, 2008a) to address potential errors or streamline analysis when it is not practical to evaluate a standard curve for every target gene. Nonetheless, it has been asserted that “the standard curve remains the most reliable and robust approach to estimate PCR assay efficiency that is broadly accepted by the community” (Svec et al., 2015) and that “the efficiency of a PCR assay is best assessed using tenfold or fivefold serial dilutions of the target nucleic acid, that is, the “Standard Curve Method” (Bustin et al., 2020).

If rigorously quantified standards with high target gene concentrations were readily available and environmental samples consistently yielded correspondingly large quantities of extracted genes, the standard curve method could be adequate—especially when the analyst is only interested in order-of-magnitude relative differences. However, this is not always the case as exemplified by sentinel surveillance of infectious diseases such as COVID-19 *via* wastewater-based epidemiology methods relying on qPCR or RT-qPCR (Chik et al., 2021; Gawlik et al., 2021). In such applications, it may be useful to be able to interpret qPCR data from samples with as few as one or two target genes per reaction or to track increases in concentration as little as 10% (*log*_10_⁡1.1≈0.04). This highlights several problems: (1) there is inherently greater variability in PCR results at low concentrations (Karrer et al., 1995), (2) there is a preponderance of non-detects at low concentrations, such as when a sampled sewershed reflects low levels of pathogen shedding and/or high levels of dilution, and (3) there is not presently a means to quantitatively describe uncertainty in qPCR-based concentration estimates to distinguish small but meaningful differences from random noise. Digital PCR (Quan et al., 2018) is an alternative to qPCR in such situations, but the question remains how to extract as much value as possible from qPCR data indicative of low concentrations of target genes when such data occur.

The greater variability of *Cq* values around the log-linear standard curve at low concentrations leads to questions about whether widely scattered data are outliers, which range of data to include in standard curve fitting, and how to interpret results indicative of low concentrations. If *Cq* values are known to inherently diverge from the log-linear pattern at low concentrations, then dismissing such divergent data as outliers is inappropriate—it discards valid data when it is the data analysis approach that is flawed. One empirical solution has been to exclude all data from highly diluted standards on account of their being outside the linear dynamic range of the calibration. Furthermore, a limit of quantification (LOQ) may be determined below which the concentration of target genes cannot be measured with acceptable precision and accuracy (Forootan et al., 2017). This also has the effect of discarding valid data when it is the data analysis approach that is flawed. Tellinghuisen and Spiess (2019) proposed a weighted least squares regression approach that mutes the contribution of low-concentration data to model fitting to account for greater variation in *Cq* values at low concentrations and improve estimation of standard curve coefficients but the method cannot be applied to standard curve data including non-detects.

Non-detects are PCR reactions resulting in no evidence of amplification, either because none occurred or there was too little amplification to reach the threshold within the completed number of cycles (McCall et al., 2014). They do not yield *Cq* values and therefore cannot be reflected in typical linear regression models. Omitting non-detects for mathematical convenience or substituting them with arbitrary *Cq* or concentration values would be a biased approach to handling non-detects in qPCR, as has been established for handling non-detects in chemistry (Helsel, 2006). Microbiological non-detects are often interpreted as censored data (e.g., concentrations below some detection limit), but Chik et al. (2018) showed the bias in applying modeling approaches tailored for analysis of censored data to microbiological non-detects that are fundamentally not censored data. In the context of qPCR, some non-detects may be right-censored *Cq* values if an insufficient number of PCR cycles was completed (e.g., *Cq* > 40 if only 40 cycles were completed), but other interpretations of non-detects must be considered. McCall et al. (2014) proposed that non-detects are missing data (amplification failures) rather than reactions lacking any target genes to amplify. PCR reactions containing target genes but failing to result in detection due to some error in preparation or execution of the PCR reaction would be indicative of a poorly controlled method (as is amplification occurring in no template controls). In contrast, reactions lacking target genes to amplify are widely recognized to occur when they are prepared from sources with low concentrations. Digital PCR is predicated on the belief that aliquots in which no amplification occurs contained no target genes (Quan et al., 2018). This leads to calculation of the gene concentration at which the observed pattern of presence–absence results is most probable (i.e., the most probable number or MPN) according to a Poisson process. Likewise, the limit of detection theory that non-detects should ideally occur 5% of the time given a standard diluted to three target genes per PCR reaction assumes that non-detects are aliquots containing no target genes according to a Poisson process (Bustin et al., 2009).

It is desirable to advance the data analysis methodology of qPCR to seamlessly describe both the linear dynamic range and the behavior of *Cq* at low concentrations—including non-detects. Rather than arbitrarily excluding valid data at low concentrations from data analysis or flagging them as unreliable, the goal should be to extract as much meaning from all data as possible. Furthermore, qPCR is currently subject to numerous guidelines aimed at generating results that are deemed reliable (e.g., Bustin et al., 2009; Ministry of the Environment, Conservation and Parks [MECP], 2021), but the degree of reliability of a given result is not quantified. For example, it should be possible to describe how precise an estimated concentration or standard curve model parameter is with interval estimates. This can be achieved through probabilistic modeling and, given the microbial context of qPCR, should align with established approaches to interpreting other types of quantitative microbiology data and the mechanisms behind observed variability (e.g., Student, 1907; McCrady, 1915; Fisher et al., 1922; Eisenhart and Wilson, 1943; Nahrstedt and Gimbel, 1996; Schmidt et al., 2022). Linear (or log-linear) regression, coupled with a parametric assumption about the distribution of residuals, is a type of probabilistic model; however, log-linear regression has chiefly been used as a means to an end in qPCR to fit a deterministic calibration curve rather than as a probabilistic model (with one exception being Tellinghuisen and Spiess, 2014). Probabilistic approaches could resolve a fundamental oversight of biostatistical qPCR models grounded in idealized theories of chemical kinetics and multiplicative effects (e.g., Rutledge and Côté, 2003; Rutledge and Stewart, 2008a; Ruijter et al., 2009; Boggy and Woolf, 2010; Svec et al., 2015)—microorganisms are not chemicals and small numbers of them (or their genes) should not be modeled as such because they are discrete objects. While it is possible to have 0.25 target genes per reaction on average among a set of replicates, it is not possible to amplify and detect a quarter of a target gene in a single reaction.

The goal of this study was to enhance standard curve modeling so that it (1) accounts for *Cq* values at low concentrations that have inflated variability and diverge from the log-linear trend, (2) seamlessly incorporates non-detects, and (3) includes uncertainty analysis for all model parameters. Integration of foundational standard curve theory supporting use of log-linear regression with probabilistic description of random errors including the Poisson-distributed initial number of target genes in each reaction was explored and led to development of an “enhanced standard curve model.” Mathematical tools are developed for model fitting and Bayesian uncertainty analysis of standard curve model parameters, and these are applied to analysis of simulated and experimental datasets. Incorporation of this modeling into qPCR-based concentration estimates and quantification of uncertainty in such estimates is beyond the scope of this model development work and analysis of standard curve data. Extensive application of the newly developed standard curve model to explore experimental design and implications upon inter-lab comparison is also beyond the scope of this work; however, the results and discussion yield practical insights that can enhance qPCR standard curve analysis, especially when the conventional log-linear model remains in everyday use.

## 2. Describing and modeling random errors in qPCR standard curve data

Conventional standard curve analysis relating *Cq* to a dilution series of standards with known concentrations is grounded in log-linear regression. Linear (or log-linear) regression is a useful statistical tool to model complex phenomena and gain empirical understanding of them in absence of detailed theory of mechanisms leading to the variability and correlation in observed data. Quantitative microbiology, however, often involves probabilistic modeling grounded in mathematical description of well-understood mechanisms leading to the random variability in observed data. This section harmonizes aspects of these two approaches to advance upon conventional standard curve modeling in qPCR with description of underlying probabilistic mechanisms. The model development is presented sequentially, beginning with rudimentary deterministic models, enfolding contemporary log-linear regression, and then embodying fundamental microbiological principles in an enhanced standard curve model that seamlessly explains behavior of qPCR at low concentrations that conventional standard curve modeling simply cannot.

### 2.1. Deterministic modeling of *Cq*

Rutledge and Côté (2003) provided a useful mechanistic derivation of the log-linear relationship in conventional standard curve modeling, though Rutledge and Stewart (2008a) subsequently applied a sigmoidal model with the goal of eliminating the need for standard curves. The derivation is summarized in this section as a starting point for model development to sequentially address some over-simplifications (sections “2.2. Log-linear regression modeling of Cq,” “2.3. Incorporating Poisson variation into qPCR data analysis,” and “2.4. Random amplification error”). This is followed by discussion of why waning amplification efficiency before reaching the fluorescence threshold does not necessarily invalidate use of the standard curve method.

If a PCR starts with *N_0_* target genes that are successively duplicated through repeated cycles, then the number of amplicons after *c* cycles is *N*_*c*_ = *N*_0_×^2*c*^. Supposing an amplicon detection threshold of *N_q_* amplicons (and equivalence to some fluorescence threshold), *Cq* is the fractional number of cycles needed to reach this number, modeled as Eq. 1 given *N*_*q*_ = *N*_0_×2*^Cq^*. Critically, a fractional number of cycles is not physically meaningful: it is a log-linear interpolation between the cycles before and after reaching the threshold. For example, if *N*_0_ = 1 and *N*_*q*_ = 1 000 000, then *Cq* is conceptually between 19 (*N*_19_ = 524 288) and 20 (*N*_20_ = 1 048 576) and can be interpolated as *Cq*≈19.93. Eq. 1 has a y-intercept (*log*_10_⁡*N*_*q*_/*log*_10_⁡2) corresponding to the number of fractional cycles needed to reach *N_q_* when *N*_0_ = 1 and a slope of approximately −3.3219 with respect to *log*_10_⁡*N*_0_.


(1) $$C\,q=\frac{\log_{10}N_{q}}{\log_{10}2}-\frac{1}{\log_{10}2}\times \log_{10}N_{0}$$

**FIGURE 1:**
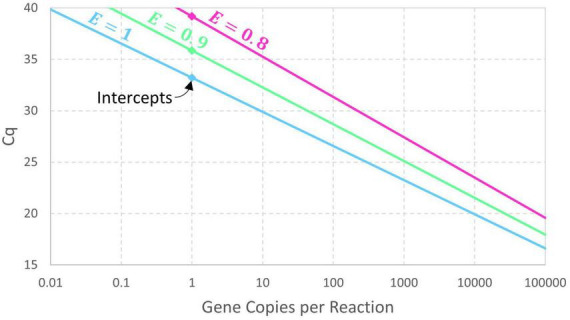
Deterministic log-linear standard curve model with amplicon detection threshold *N*_*q*_ = 10^10^ and amplification efficiency *E* having values of 1, 0.9, and 0.8. The corresponding intercept values are 33.22, 35.87, and 39.17, respectively.

The presumption of perfect doubling of amplicons in each cycle may be generalized by adding a parameter for amplification efficiency (*E*) so that cycles initially achieve (1 + *E*)-fold amplification. If exponential amplification persists for at least *Cq* cycles, then *N*_*q*_=*N*_0_×(1 + *E*)^*Cq*^ and this can be rearranged as Eq. 2. Expressing this model in terms of an intercept and slope yields the conventional log-linear standard curve (Eq. 3). An estimate of amplification efficiency is commonly back-calculated from the slope using *E* = 10^−1/*Slope*^−1, assuming precisely controlled standards and dilutions. Reducing the exponential amplification efficiency increases the intercept (by increasing the number of cycles needed to reach *N_q_*) and leads to a slightly steeper slope as shown in Figure 1. These equations are deterministic because there is no random variation in the value of *Cq* corresponding to a particular value of *N_0_*.


(2) $$C\,q=\frac{\log_{10}N_{q}}{\log_{10}\left(1+E\right)}-\frac{1}{\log_{10}\left(1+E\right)}\times \log_{10}N_{0}$$

(3) $$C\,q=I\,n\,t\,e\,r\,c\,e\,p\,t+S\,l\,o\,p\,e\times \log_{10}N_{0}$$

Although amplification efficiency is known to wane eventually, leading to the plateauing of qPCR fluorescence curves, the persistence of exponential growth until the threshold has been disputed (Rutledge and Stewart, 2008b). However, this assumption is not strictly necessary for the above model and less restrictive mechanistic criteria may be described. Separation of *Cq* values must be established by initial exponential growth persisting until the number of amplicons is greater than the largest tested initial number of target genes (*N_0_*). If this is not the case, waning amplification efficiency will lead to reduced separation of *Cq* values at high concentrations and an upper limit on the linear dynamic range. At higher numbers of amplicons, the separation in *Cq* values established by exponential amplification is retained so long as all curves have the same shape—even if waning amplification efficiency leads to sub-exponential amplification. The fluorescence threshold can be set within this sub-exponential region, but not so high that divergence toward reaction-specific plateaus affects the separation of *Cq* values. Within this region, *Cq* is not necessarily the number of cycles of exponential growth needed to get to the threshold; the intercept behaves as a tuning parameter to quantify the number of cycles required to amplify a single target gene to the fluorescence threshold whether all of those cycles featured exponential amplification or not.

### 2.2. Log-linear regression modeling of *Cq*

The need to analyze standard curve data using a probabilistic approach rather than a deterministic one is exemplified by variation in *Cq* values among replicates at a particular standard concentration. Log-linear regression is widely used and supported by the log-linear deterministic model in the section “2.1. Deterministic modeling of Cq,” but random scatter in *Cq* values about the fitted line and its implications are rarely addressed. This section emphasizes potential mechanisms and modeling of this variation. Presuming that the initial number of target genes (*N_0_*) is known and that amplification proceeds deterministically until *Cq*, error can arise from how *Cq* is determined from fluorescence data or from well-to-well variation in amplification efficiency. Determination of *Cq* from fluorescence data involves evaluating background fluorescence for each well and potentially each cycle and using relative fluorescence measurements to represent the portion of the fluorescence attributed to amplifying target genes. Normalization may also be used to correct for well-to-well variation in the capacity to detect fluorescence. Any imprecision in individual fluorescence readings or how they are adjusted would affect the precision of the relative fluorescence readings between which *Cq* is interpolated. Variation in amplification efficiency could contribute to scatter in *Cq* values because reactions with lower amplification efficiency would take longer to reach the threshold. However, the effect could diminish at higher concentrations as less amplification is needed to reach the threshold. These errors are mechanistically described as “*Cq* residual error” because deviations from a fitted regression model are called residuals.

If the error in determining *Cq* for each well is normally distributed with mean zero and standard deviation σ (called “*Cq* residual standard deviation” herein), then the result is the conventional log-linear regression model often used in standard curve fitting together with mechanistic interpretation of the random error component. Specifically, Eq. 4 builds probabilistically upon Eq. 3 using *N*(μ,σ^2^) notation for a normal distribution with mean μ and variance σ^2^. This model carries on with constant variance σ^2^ to values of *N_0_* much less than 1 target gene per reaction and cannot explain non-detects that do not yield *Cq* values at all.


(4) $$C\,q∼N\,\left(I\,n\,t\,e\,r\,c\,e\,p\,t+S\,l\,o\,p\,e\times \log_{10}N_{0},\sigma^{2}\right)$$

### 2.3. Incorporating Poisson variation into qPCR data analysis

The log-linear regression standard curve model (Eq. 4) relates *Cq* to an integer initial number of target genes (*N_0_*) rather than a standard concentration λ. The discordance in contemporary qPCR theory associated with assuming *N*_0_ = λ has diminishing effect at high concentrations, which is why conventional log-linear regression works well in a linear dynamic range but not at low concentrations. At low concentrations, random variation (random sampling error) in the number of discrete objects in an aliquot of known volume from a source of known concentration becomes important. Assuming that volume is carefully controlled, that standards are precisely quantified and diluted, and that target genes are randomly dispersed in these standards, this variation should follow a Poisson distribution (Eq. 5). This error is denoted herein as “reaction random sampling error” because it applies to the initial number of target genes in a PCR reaction. A separate form of random sampling error describing variation in the integer number of instances of the target gene contained in a sample collected from the environment (e.g., prior to processing and extraction) is outside the scope of this work.


(5) $$N_{0}∼P\,o\,i\,s\,s\,o\,n\,\left(\lambda \right)$$

This variation is sometimes acknowledged in qPCR data analysis literature, but it is generally not reflected in approaches to standard curve fitting. The weighted regression approach of Tellinghuisen and Spiess (2019) suppresses excess variation at low standard concentrations due to this error but does not account for non-detects (*N*_0_ = 0) that cannot yield *Cq* values and therefore cause the function *Cq* = *a* + *b*×*log*⁡*N*_0_ to not have a variance with which to assign weights. Their variance formula is accurate for concentrations at which non-detects are improbable (e.g., >7 gc/rxn) if a zero-truncated Poisson distribution is assumed, but it misrepresents the effect of Poisson variation at lower concentrations. Rather than using the idea of Poisson-distributed *N_0_* to develop a patch to least squares regression, this research formally conditions a probabilistic regression model (Eq. 4) on Poisson-distributed *N_0_* (Eq. 5) using hierarchical probabilistic modeling. Addressing the discrete nature of genes as well as the Poisson-distributed variation in the initial number of target genes contained in an aliquot of diluted standard leads to an enhanced standard curve model. All random errors that pertain to preparation of environmental samples for qPCR but not to standard curve experiments using standards with extracted or synthetic genes are outside the scope of this work. This model reflects non-detects (*Cq* = *ND*) arising from an *N_0_* of zero but not from amplification failures or running an insufficient number of cycles.

**FIGURE 2:**
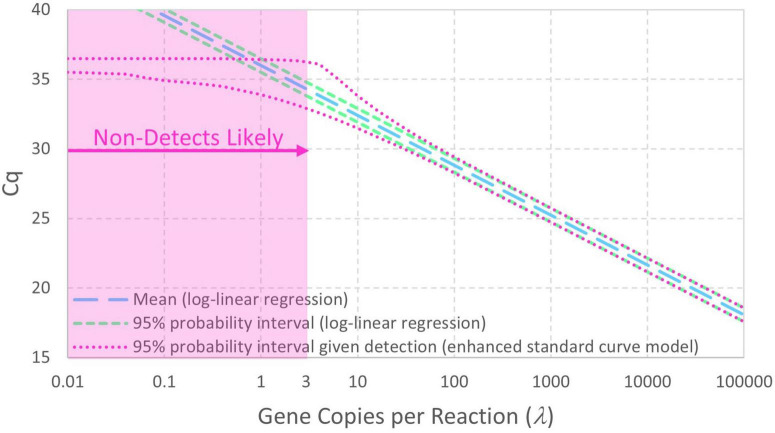
Comparison of standard curve models with and without Poisson-distributed reaction random sampling error given intercept 36, amplification efficiency *E* = 0.9, and *Cq* residual standard deviation σ = 0.25. Non-detects are illustrated as likely below 3 gc/rxn, and the 95% probability interval for the enhanced standard curve model (with reaction random sampling error) is conditional on detection.

Figure 2 contrasts 95% probability intervals of *Cq* (conditional on detection) as a function of continuous concentrations of gene copies per reaction (gc/rxn) with and without reaction random sampling error. The contemporary log-linear regression standard curve model goes off to concentrations below 1 gc/rxn with homogeneous variance and cannot explain non-detects. Reaction random sampling error, on the other hand, leads to (1) increased variability of *Cq* values at lower concentrations, (2) wells initially containing zero target genes that cannot yield *Cq* values and are therefore non-detects, and (3) *Cq* values not being able to greatly exceed the intercept. The effect of this random sampling error appears below 30 gc/rxn in this example, but it would be evident at higher concentrations with smaller values of *Cq* residual standard deviation σ. With equal numbers of technical replicates at each tested concentration, non-detects lead to progressive sparsity of numeric data below about 3 gc/rxn. *Cq* values cannot greatly exceed the intercept because *N*_0_ = 1 is the smallest non-zero initial number of target genes.

**FIGURE 3:**
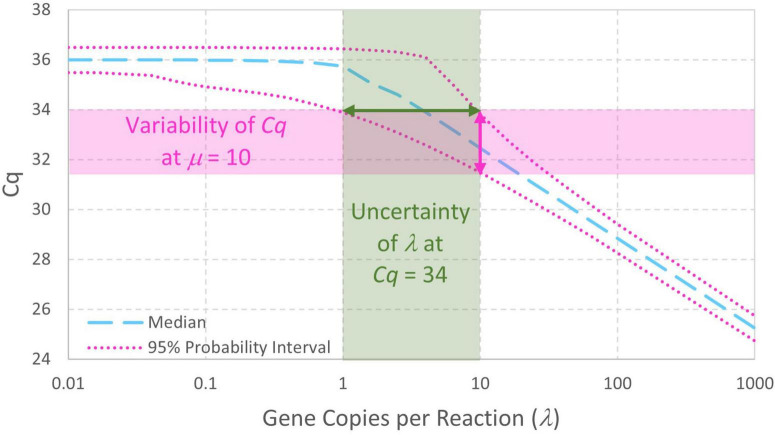
Probability intervals describing variability in *Cq* as a function of gene copies per reaction also give a visual approximation to begin quantitatively understanding the uncertainty in qPCR-based concentration estimates. The displayed standard curve model includes Poisson-distributed reaction random sampling error and has intercept 36, amplification efficiency *E* = 0.9, and *Cq* residual standard deviation σ=0.25.

The enhanced standard curve model combining Eqs. 4, 5 facilitates analysis of standard curve data without arbitrary decisions about which *Cq* values arising from low standard concentrations should be included in standard curve model fitting. It also facilitates simulation of standard curve data (section “3.1. Simulation of standard curve data”), straightforward evaluation of probabilities and parameter estimates (section “3.2. Numerical integration to compute probability intervals or maximum likelihood estimates”), and evaluation of uncertainty in fitted standard curve model parameters (section 3.3. Parametric uncertainty analysis of standard curve parameters”). In addition to enabling computation of the variability in *Cq* given a standard concentration λ (in gc/rxn), this model is a first step toward evaluating the uncertainty in qPCR-based concentration estimates (Figure 3). Notably, this model includes essentially the same parameters as log-linear regression (e.g., intercept, slope, and the largely unreported residual standard deviation σ). Usually, increasing the complexity of a model increases the number of parameters and the need for supporting data. No parameters are added in this case because it replaces the assumption that *N*_0_ = λ with a Poisson distribution having only one parameter (λ).

### 2.4. Random amplification error

Like the initial number of target genes in a reaction (*N_0_*), the number of amplicons after *c* PCR cycles (*N_c_*) must also be a non-negative integer. For example, if *N*_0_ = 1 and *E* = 0.9, then a 1.9-fold increase is expected in each cycle, but it is impossible to have 1.9 amplicons after the first cycle. Instead, there would be a 90% chance of amplifying to two amplicons and a 10% chance of remaining with only one, which yields 1.9 amplicons on average. If this solitary target gene in the well did not amplify in the first cycle, it is as if the first cycle did not exist in terms of advancing toward detection, so *Cq* is raised by one cycle. The number of amplicons after *c* cycles may be modeled recursively as a discrete-time Markov chain initialized with *N_0_* and the transition matrix for cycle *c* can be populated using Eq. 6. Modeling this “random amplification error” using a binomial distribution presumes that each target gene present either duplicates or fails to duplicate with probability equal to amplification efficiency *E* that cannot exceed 100%. The resulting value of *Cq* may then be modeled using Eq. 7 given a value of *c* corresponding to a large value of *N_c_* that remains within the exponential phase. Addition of this error adds no parameters to the standard curve model because each binomial distribution depends only on the already specified input *N*_*c–1*_ and amplification efficiency *E* that was already in the model.


(6) $$N_{c}-N_{c-1}∼b\,i\,n\,o\,m\,i\,a\,l\,\left(N_{c-1},E\right)$$

(7) $$C\,q-c∼N\,\left(I\,n\,t\,e\,r\,c\,e\,p\,t+S\,l\,o\,p\,e\times \log_{10}N_{c},\sigma^{2}\right)$$

**FIGURE 4:**
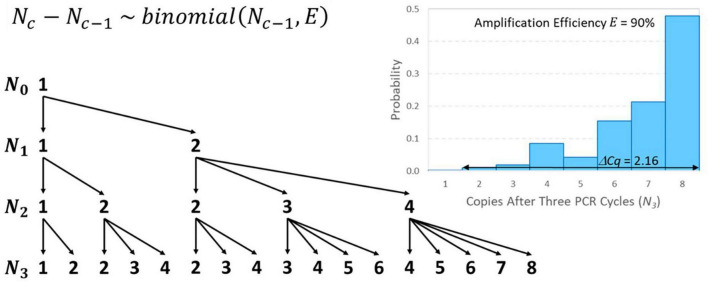
Tree diagram illustrating all possible outcomes of amplifying *N*_0_ = 1 target gene through three PCR cycles and a distribution of resulting numbers of amplicons after three cycles with *E* = 90% amplification efficiency. The conditional probability associated with each arrow may be calculated using the binomial distribution shown. In terms of 1.9-fold amplification, the difference between having 2 or 8 amplicons after three cycles is 2.16 *Cq* on average.

Figure 4 provides an illustrative example of random amplification error with *N*_0_ = 1, *E* = 0.9, and three cycles (because algebraically exploring the effect of random amplification error with numbers of cycles typical of qPCR is intractable). The mean number of amplicons after three cycles is 6.859 (i.e., 1.9^3^) as expected, but there is substantial random variation. There is a 47.8% chance that there will be 8 amplicons after three cycles and a 1% chance that there will only be two amplicons. The difference between these outcomes in terms of *Cq* is 2.16 because 2 × 1.9^2.16^ = 8. Random amplification error diminishes as the initial number of target genes or number of amplicons increases, meaning that it becomes trivial for larger values of *N_0_* and, in the *c^th^* cycle, for large *N*_*c–1*_. Thus, its effect on variability in *Cq* is similar to reaction random sampling error in that it is relatively inconsequential at high concentrations and becomes progressively more important at low concentrations. For example, three cycles with *N*_0_ = 10 and *E* = 0.9 are likely to yield between 57 and 78 amplicons (not shown), which would have an effect of only 0.49 on the value of *Cq*. This is related to the diminishing relative standard deviation (*RSD*) of the binomial distribution in Eq. 6 ($R\,S\,D=\sqrt{\left(1-E\right)/\left(N_{c-1}\times E\right)}$) as *N*_*c–1*_ increases.

### 2.5. Additional random errors affecting standard curve data

The foregoing development of an enhanced standard curve model and its ensuing application are grounded in the assumption that *N*_0_∼*Poisson*(λ) and that λ is precisely known. Specifically, it focuses on modeling the relationship between *Cq* and the nominal concentration of gene copies per reaction (λ) but does not address the accuracy of λ or validity of the Poisson distribution describing random sampling error in wells. Noting that assumptions and limitations are central to model development, additional error mechanisms that are not modeled herein are described below.

There are many mechanisms through which λ may be imprecisely known, including (1) error in the nominal concentration of the undiluted standard, (2) volumetric error in the dilution series, and (3) losses in the dilution series. All of these errors lead to biased concentration estimates. If the concentration of the undiluted standard is higher than indicated, *Cq* would be reduced and non-detects would be less common than anticipated at nominal concentrations near and below 1 gc/rxn. Conversely, if the concentration of the undiluted standard is lower than indicated, *Cq* would be increased and non-detects may become unexpectedly common at nominal concentrations near and above 1 gc/rxn. Systematic dilution errors would lead to a compounding error that makes the nominal concentration of more diluted standards more inaccurate (e.g., if a nominal 2-fold dilution is actually a 1.9-fold or 2.1-fold dilution). Consistent losses among dilutions (e.g., due to microorganisms or target genes not successfully discharged from the pipette tip) would also lead to a compounding error akin to dilution error. For example, a 2-fold dilution with 2% losses is effectively a 2.04-fold dilution (2/0.98). Over-dilution and losses will lead to nominal concentrations that are over-stated, increase *Cq* values, decrease amplification efficiency estimates, and lead to unexpectedly common non-detects near and above 1 gc/rxn. Conversely, under-dilution leads to nominal concentrations that are under-stated, decreases *Cq* values, increases amplification efficiency estimates, and leads to unexpectedly rare non-detects near and below 1 gc/rxn. It is therefore important to regularly ensure accurate calibration of pipettes to prevent biased estimation of PCR efficiency and associated quantification of target genes arising from insufficiently controlled standard curve data. Unknowingly using an inadequately calibrated pipette or misusing a pipette functionally calibrates qPCR to improperly quantified standards so that both absolute and relative quantification would be biased.

The assumption of a Poisson distribution for well-random sampling error (Eq. 5) is firmly grounded in theory (Student, 1907), but extraneous sources of variation can lead to over-dispersion (Schmidt et al., 2014). These can include spatial heterogeneity in the standard (poor mixing) or non-constant losses or volumetric error in the transfer to the well. A final potential cause of over-dispersion would be clustering of target genes. Target genes that are bound in groups within an otherwise homogeneous and accurately quantified source (i.e., not just having spatially varying concentration due to poor mixing) are known to invalidate the Poisson assumption. This can inflate variability in *N_0_* relative to a Poisson model, thus increasing the variability in *Cq* values and increasing the probability of non-detects at specific concentrations. In the analogous context of dose–response models (Schmidt, 2015) in which doses are assumed to be accurately quantified and aggregates are assumed to break up following consumption, clustering would create an illusion that a pathogen is less infectious than it really is by reducing the probability of infection for specific doses. This issue could compromise quantitative reliability of digital PCR because MPN calculations used to interpret arrays of presence–absence results are usually predicated on a Poisson model. Presuming that these issues can be adequately addressed in well-controlled standard curve experiments, modeling of over-dispersion with respect to the Poisson distribution is outside the scope of this work.

## 3. Methods for using the developed probabilistic models in simulation and model-fitting

Following the model development discussion in the section “2. Describing and modeling random errors in qPCR standard curve data” that focused on representing the foundational theory of standard curve modeling and adapting it to reflect physically meaningful random error mechanisms, this section addresses the more mathematical topic of how to use these models. Tasks addressed include simulation of standard curve data, computation of probability intervals, model fitting using maximum likelihood estimation, and Bayesian methods to evaluate parameter uncertainty.

### 3.1. Simulation of standard curve data

In mechanistically derived models, Monte Carlo simulation can be useful to explore the anticipated effects of various error mechanisms or to contrast alternative experimental designs or data analysis approaches. The models developed in the section “2. Describing and modeling random errors in qPCR standard curve data” can be used to simulate *Cq* values given values of model parameters *Intercept*, *E*, and σ as well as concentration λ. A custom function in R (R Core Team, 2020) with which data can be simulated is provided in the Supplementary content along with an illustrative example of its use.

### 3.2. Numerical integration to compute probability intervals or maximum likelihood estimates

To compute probability intervals graphically illustrating variability in *Cq* values or fit model parameters to available data using maximum likelihood estimation, it is necessary to algebraically characterize the distribution of *Cq*. Reflecting reaction random sampling error in the log-linear regression model adds complexity to evaluation of the distribution of *Cq*. Specifically, the model becomes hierarchical because Eq. 4 is conditional on a value of *N_0_* that is also random according to Eq. 5. Computing the unconditional (or marginal) distribution of *Cq* requires summation of all non-zero values of *N_0_* and their respective probabilities, which must be done numerically. The resulting probability density function (Eq. 8) describes only the numeric values of *Cq* while Eq. 9 describes the non-numeric outcome that *Cq* is undetermined when *N*_0_ = 0, resulting in a non-detect (*Cq* = *ND*). The cumulative distribution function (Eq. 10) is calculated using the standard normal cumulative distribution function Φ(.) and has a maximum value of 1−*e*^−λ^ due to non-detects. Derivation of these equations is provided in the Supplementary content, as is an R function with which to perform these calculations. Consideration of random amplification error in this study is limited to theoretical development and simulation because numerical integration is intractable.


(8) $$f\,\left(C\,q\right)=\sum_{N_{0}=1}^{\infty }\frac{e^{-\lambda }\,\lambda^{N_{0}}}{N_{0}!}\times \frac{1}{\sqrt{2\,\pi }\,\sigma }\exp\left\{-\frac{{\left[C\,q-\left(I\,n\,t\,e\,r\,c\,e\,p\,t+S\,l\,o\,p\,e\cdot \log_{10}N_{0}\right)\right]}^{2}}{2\,\sigma^{2}}\right\}$$

(9) $$P\left(Cq=ND\right)=P\left(N_{0}=0\right)=e^{-\lambda }$$

(10) $$F\,\left(C\,q\right)=\sum_{N_{0}=1}^{\infty }\frac{e^{-\lambda }\,\lambda^{N_{0}}}{N_{0}!}\times \text{Φ}\,\left(\frac{C\,q-\left(I\,n\,t\,e\,r\,c\,e\,p\,t+S\,l\,o\,p\,e\cdot \log_{10}N_{0}\right)}{\sigma }\right)$$

Equation 10 is useful to compute probability intervals for *Cq*, particularly if it is made conditional on detection by dividing it by 1−*e*^−λ^. A 95% probability interval such as the ones displayed in Figures 2, 3 can be calculated for each of a range of concentrations by determining the *Cq* values corresponding to 2.5% and 97.5% cumulative probability. Eq. 8 is useful for maximum likelihood estimation to determine the values of model parameters that maximize the probability of a set of observed data (R scripts are provided in the Supplementary content). Because non-detects arising from reactions containing no target genes depend on concentration λ (Eq. 9) but not on properties of the PCR, they have no effect on estimation of the fitted standard curve model parameters (i.e., they appear only as constants in the likelihood function used for inference of standard curve model parameters). Non-detects may therefore be omitted from standard curve fitting (but *not* analysis of environmental data) because it is mathematically justifiable to omit them. This differs from just arbitrarily omitting them because they are incompatible with log-linear regression.

### 3.3. Parametric uncertainty analysis of standard curve parameters

Ideally, science should not be based solely on providing estimates of values inferred from data but should indicate how good the estimates are or what range of other values could be supported by the data. Parametric uncertainty in estimated parameters of probabilistic models may be fully represented using Bayesian Markov chain Monte Carlo (MCMC) and software such as OpenBUGS (3.2.3 rev 1012; Lunn et al., 2000). The Bayesian process merges observed data and subjective beliefs about parameters (represented by prior distributions) to express uncertainty in the values of estimated model parameters in the form of a posterior distribution. Relatively uninformative priors are often used in absence of well supported subjective beliefs so that the posterior distribution is most strongly influenced by the objective information from the data. Because posterior distributions are often difficult to evaluate algebraically, MCMC is used to draw a set of values representing this posterior distribution. The 2.5th and 97.5th percentiles of the generated parameter values can then be used to define a 95% credible interval in which the analyst is entitled to believe that the true value of the parameter is contained with 95% chance. This section details Bayesian analysis of uncertainty in the parameters of the enhanced standard curve model applying MCMC in OpenBUGS (see model code in Supplementary content). Random amplification error was not implemented in OpenBUGS due to limitations in the model specification step that precluded incorporation of Eq. 6.

Analyses performed herein included a log-uniform prior on the amplicon detection threshold (0 < *log*_10_⁡*N*_*q*_ < 15), a uniform prior on amplification efficiency (0 < *E* < 1), a log-uniform prior on the *Cq* residual standard deviation (−5 < *log*_2_⁡σ < 1). The upper bound for *N_q_* of 10^15^ corresponds to approximately 50 two-fold amplifications of a single target gene. This relatively uninformative prior favors smaller values of *N_q_* but corresponds to a uniform prior on the intercept (conditional on a particular value of amplification efficiency *E*). Restricting amplification efficiency to *E* < 1 reflects that it is not theoretically possible to amplify a single target gene more than once in a single PCR cycle and is otherwise relatively uninformative. This prior can have a substantial effect on estimation of *E* when conventional log-linear regression would give an estimate near or above 100%. Dilution errors (too little diluent and/or pipetting excess standard) and inhibition at high concentrations are known mechanisms for amplification efficiency estimates exceeding 100%. The prior on *Cq* residual standard deviation (σ) slightly favors smaller values, but it is wide enough to be relatively uninformative in most cases. *Cq* values typically vary to some extent at high concentrations and a standard deviation of *Cq* values below 0.5 has been proposed as a performance criterion (Ministry of the Environment, Conservation and Parks [MECP], 2021). Default updating algorithms were used with a burn-in of 10,000 iterations and thinning to every 100th of the next 100,000 iterations to ensure that the generated sample provided a good representation of the posterior distribution. History plots indicated rapid convergence and excellent mixing, and each analysis took about 2 min.

## 4. Application of model to analysis of simulated standard curve data

For illustrative purposes, an analysis was carried out with 101 data simulated (i.e., randomly generated using the developed models) using concentrations that are equally spaced in logarithmic scale between 0.01 and 1000 gc/rxn (Supplementary Table 1). Simulation of realistic dilutions with many technical replicates at each dilution has less illustrative value because of overlapping points in plots. Data were simulated both with and without random amplification error using an intercept of 36, amplification efficiency *E* = 0.90, and *Cq* residual standard deviation σ=0.25. The same Poisson-distributed values of *N_0_* and normally distributed *Cq* residual error were used in each scenario so that results differ only in the inclusion or exclusion of random amplification error. The purpose of this simulation experiment was to (1) illustrate the effect of random amplification error relative to only reaction random sampling error and (2) compare alternative model fitting techniques.

**FIGURE 5:**
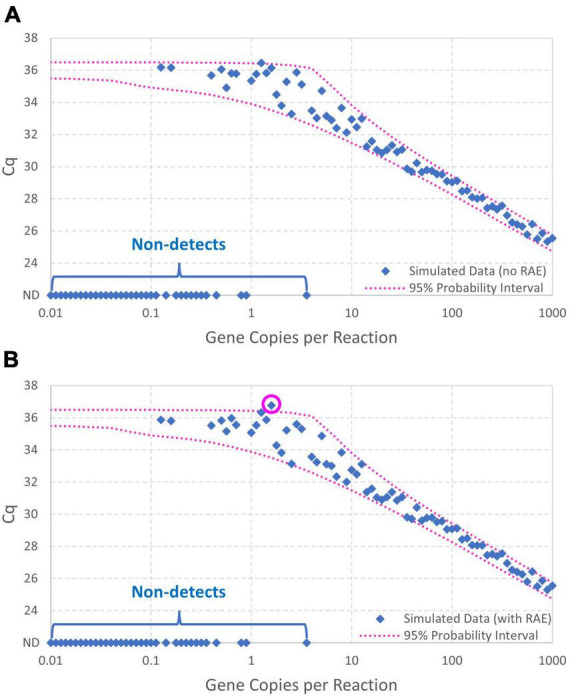
Data simulated **(A)** with and **(B)** without random amplification error (RAE). Data were generated using intercept 36, amplification efficiency *E* = 0.9, and *Cq* residual standard deviation σ=0.25, and the 95% probability interval was calculated using the same model without including random amplification error. An extreme *Cq* value highlighting effects of random amplification error is circled.

Figure 5A shows the data simulated without random amplification error (with non-detects plotted as *Cq* = *ND* to allow their illustration). Figure 5B likewise shows the data simulated with random amplification error. Both figures show the 95% probability interval for *Cq* conditional on detection computed using the model without random amplification error. The similarity of the two graphs shows the subtlety of random amplification error relative to reaction random sampling error, with differences almost imperceptible above 10 gc/rxn. By chance, only one observation falls outside the 95% probability interval in each figure, which is not a particularly improbable result for 67 detections. In Figure 5B, however, the simulated *Cq* value at 1.58 gc/rxn (circled) is well above the upper bound of the probability interval. This datum had *N*_0_ = 1, and non-amplification of a solitary target gene in the first cycle is known to raise *Cq* substantially. The probability of such a high *Cq* value given only reaction random sampling error is < 0.001.

Three alternative parameter estimation approaches were applied to each simulated dataset for illustrative purposes, with results summarized in Table 1. These included (1) log-linear regression with all obtained *Cq* values, (2) log-linear regression with only obtained *Cq* values at concentrations above the highest concentration with a non-detect, and (3) maximum likelihood estimation using the enhanced standard curve model (which does not reflect random amplification error).

**TABLE 1:** Comparison of estimates of the intercept, amplification efficiency (*E*) and *Cq* residual standard deviation (σ) using alternative model fitting approaches and data simulated with and without random amplification error.

Modeling approach	Figure 5A data (no amplification error)	Figure 5B data (with amplification error)
Model used for simulation (with or without random amplification error)	*Intercept* = 36.00 *E* = 0.9000 σ=0.2500	*Intercept* = 36.00 *E* = 0.9000 σ=0.2500
Approach 1: Log-linear regression omitting non-detects	*Intercept* = 35.42 *E* = 1.0328 σ = 0.7181 *R*^2^ = 0.9569	*Intercept* = 35.38 *E* = 1.0457 σ=0.7769 *R*^2^ = 0.9490
Approach 2: Log-linear regression including only *Cq* values for concentrations above highest non-detected gc/rxn	*Intercept* = 35.95 *E* = 0.9179 σ=0.4138 *R*^2^ = 0.9744	*Intercept* = 36.02 *E* = 0.9084 σ=0.4319 *R*^2^ = 0.9726
Approach 3: Maximum likelihood estimation using enhanced standard curve model	*Intercept* = 35.98 *E* = 0.9124 σ=0.2433	*Intercept* = 35.93 *E* = 0.9214 σ = 0.2984

Critically, when log-linear regression was fit to all *Cq* data with non-detects omitted (approach 1), the intercept was under-estimated and both the amplification efficiency and *Cq* residual standard deviation were substantially over-estimated. Applying log-linear regression to *Cq* values at inappropriately low concentrations can lead to amplification efficiency estimates >100% as shown. This result was anticipated because the regression is being applied to data including *Cq* values at concentrations below 1 gc/rxn that depart markedly from the log-linear trend. One approach to eliminate this effect is to only apply log-linear regression to *Cq* values from dilutions with concentrations above the highest concentration at which a non-detect was obtained (approach 2). This approach yields more appropriate intercept and amplification efficiency estimates by excluding any non-linearity, but the *Cq* residual standard deviation is still over-estimated because log-linear regression cannot explain increasingly variable *Cq* values as the concentration nears 1 gc/rxn. Values of the coefficient of determination (*R*^2^) are shown for the regression methods, as recommended (Bustin et al., 2009). The *Cq* residual standard deviation (σ) quantifies the consistency of *Cq* values obtained at high concentrations, while *R*^2^ quantifies the linearity of the collection of data. Notably, *R*^2^ can be a misleading performance metric when comparing standard curves with different ranges of tested concentrations: a wider range with poorer consistency of *Cq* values may yield a higher *R*^2^ value than a narrower range with better consistency of *Cq* values.

Logically, maximum likelihood estimation with the enhanced standard curve model (approach 3) yields some of the best parameter estimates because it applies the model used to simulate the data in reverse (except for exclusion of random amplification error). Reversibility is a key feature of probabilistic models that allows them to be used to simulate data given parameters or estimate parameters given data (Schmidt et al., 2022). This method yields the lowest estimates of *Cq* residual standard deviation (σ) because it accounts for the excess variation in *Cq* at low concentrations that is introduced by reaction random sampling error. The maximum likelihood estimates for the Figure 5B data include a higher estimate of the *Cq* residual standard deviation than for the Figure 5A data, possibly to accommodate the inflated variation in *Cq* and one particularly high value attributed to random amplification error.

**FIGURE 6:**
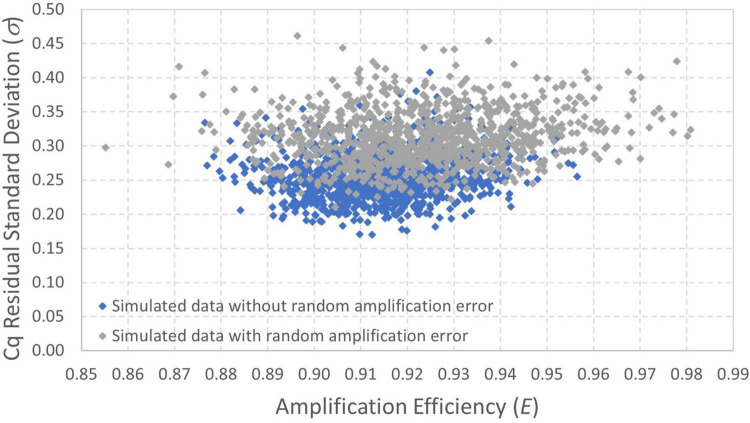
Scatterplot of posterior distributions quantifying uncertainty in amplification efficiency and *Cq* residual standard deviation estimated from datasets simulated with and without random amplification error (Figures 5A,B, respectively). The data were simulated with *E* = 0.9 and σ=0.25.

Bayesian Markov chain Monte Carlo was also applied to both simulated datasets. Figure 6 shows a scatterplot of results for amplification efficiency *E* and *Cq* residual standard deviation σ because these are the two parameters for which the maximum likelihood estimates differ the most between the datasets with and without random amplification error (Table 1). These results show that the amplification efficiency with which the data were simulated (*E* = 0.9) is well within the quantified uncertainty for each dataset. There is, however, particular divergence in estimation of the *Cq* residual standard deviation (σ = 0.25). Analysis of data simulated with random amplification error using a statistical procedure that does not include it leads to over-estimation of σ. However, this approach is less biased than the conventional log-linear regression model (Table 1). Continued work to incorporate random amplification error into data analysis and model fitting may be warranted to resolve this bias.

## 5. Application of model to analysis of experimental standard curve data

Standard curves may be evaluated for two reasons: to evaluate performance metrics such as a limit of detection or limit of quantification or to provide calibration that facilitates estimation of the concentration of samples that were not prepared from standards. Standard curves targeting evaluation of a limit of detection or limit of quantification may include large numbers of technical replicates prepared from dilutions with low concentrations (sometimes below 1 gc/rxn) but do not always include high concentrations. Standard curves prepared for plate-specific calibration may have fewer technical replicates and a wide range of concentrations that may not include concentrations near 1 gc/rxn. To provide a useful illustrative example reflecting both extremes, a standard curve of the N1 region of the SARS-CoV-2 nucleocapsid gene using a standard with synthetic RNA transcripts (#COV019, EDX, USA) was prepared. Specifically, the standard was serially diluted (2x) in 20 ng/μL Poly(A) (#10108626001, Roche, Germany) and TE buffer (#BP2473100, Fisher Scientific, USA) with concentrations ranging from 200 to 0.39 gc/rxn. The assay for the N1 target followed the CDC 2019-nCoV Real-Time RT-PCR Diagnostic Panel (Centers for Disease Control and Prevention [CDC], 2020) with primers and probes purchased from Sigma-Aldrich (USA). TaqPath™ 1-Step RT-qPCR Master Mix, CG (A15299, ThermoFisher, USA) was used. Nine technical replicates for each standard and six no-template controls (NTCs) were plated on a 96-well plate (Supplementary Table 2). qPCR was run on the OPUS system (Bio-Rad, USA) with conditions outlined in Supplementary Table 3. Based on the developed model it was anticipated that there would be (1) low variability in *Cq* at high concentrations, (2) increasing variability in *Cq* at low concentrations, (3) non-detects starting to appear near 3 gc/rxn, (4) some *Cq* values deviating from the log-linear pattern below 1 gc/rxn, and (5) no *Cq* values more than perhaps 3σ above the intercept.

**FIGURE 7:**
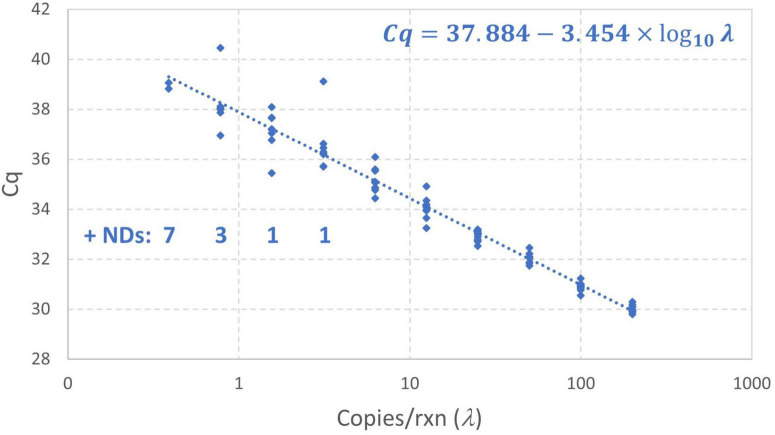
Empirical standard curve data for synthetic N1 target of SARS-CoV-2 using nine technical replicates at each two-fold dilution between 0.39 and 200 gc/rxn. The log-linear regression standard curve model fitted by the instrument software is shown.

Figure 7 shows the results of this standard curve experiment as well as the log-linear regression standard curve model fit by the instrument software to all *Cq* values (omitting only the non-detects). The *Cq* values at concentrations below 1 gc/rxn do not clearly diverge from the log-linear trend and there are several data that are more than 1 cycle above the intercept of 37.88 obtained using conventional log-linear regression omitting non-detects. Such results may be attributable to the small number of detections at low concentrations and excess variation due to random amplification error. Maximum likelihood estimation was applied to these data using the enhanced standard curve model. The resulting parameter estimates were an intercept of 38.35, amplification efficiency of 0.8593, and *Cq* residual standard deviation of 0.4363. In contrast, the parameter estimates obtained by log-linear regression with non-detects omitted are 37.88, 0.9476, and 0.5899, respectively. This analysis flagged the datum with *Cq* = 40.46 as an extreme value (because the modeled probability of a *Cq* higher than this at 0.78 gc/rxn was <10^–6^). Small numbers of wells yielding *Cq* values that are difficult to explain are not uncommon in practice, and it is desirable to provide a statistical basis for excluding such results in absence of a known error. The analysis was repeated with this value excluded, leading to an estimated intercept of 38.18, amplification efficiency of 0.8906, and *Cq* residual standard deviation of 0.3072. The latter parameter was particularly affected by excluding the datum in question.

**FIGURE 8:**
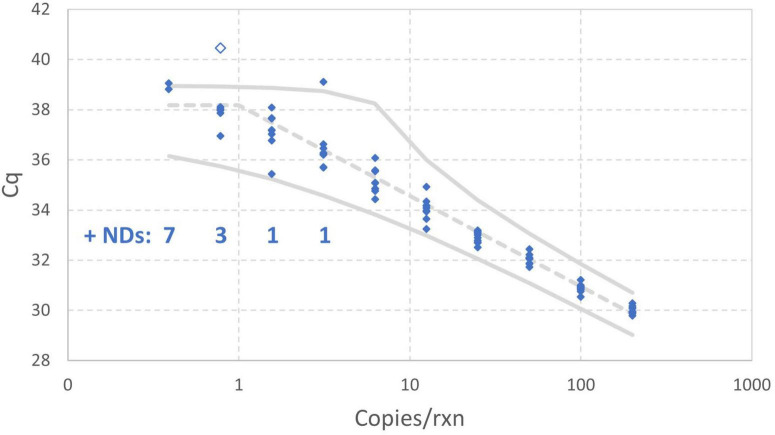
Empirical standard curve data for synthetic N1 target of SARS-CoV-2 using nine technical replicates at each two-fold dilution between 0.39 and 200 gc/rxn. 95% probability intervals (solid gray) and the median (dashed gray) were computed using maximum likelihood estimates for the enhanced standard curve model with one datum excluded as an extreme value.

Figure 8 includes 95% probability intervals calculated using the maximum likelihood estimates of the model parameters with the one extreme datum excluded. With these probability intervals, it is evident that there are a few somewhat improbable *Cq* values, but none are so glaring as the one datum that was excluded. Bayesian Markov chain Monte Carlo analysis (not shown) of the data excluding the one high *Cq* value was carried out to quantify uncertainty in each estimated parameter of the enhanced standard curve model using 95% credible intervals: the results are an intercept of 38.18 (37.95–38.40), amplification efficiency of 89.06% (84.93%–93.79%), and *Cq* residual standard deviation of 0.3072 (0.2533–0.4038).

## 6. Discussion

Quantitative inference about target gene concentrations *via* qPCR has often been grounded in log-linear regression to establish a standard curve. This borrows concepts from analytical chemistry, which often depends on linear calibration models to relate observed signals to concentrations of interest. Within a linear dynamic range comprised exclusively of relatively high concentrations that do not generate non-detects or particularly inflated variability in *Cq*, this approach is dependable. However, conventional standard curve analysis requires exclusion of problematic data at low concentrations (e.g., non-detects and data exhibiting excess variation in *Cq* or non-linearity) for model fitting. There is also a lack of trust in extrapolation of the standard curve to high *Cq* values and lack of clarity about handling of non-detects when it is applied to estimate concentrations in environmental samples. In many cases it is easier to dismiss these “non-standard” data than to tackle the statistical problem of inference from data that the log-linear regression model cannot explain. However, scenarios where low concentrations abound and small changes in concentration (e.g., 10% rather than an order of magnitude) are important—such as wastewater-based epidemiology for SARS-CoV-2 (e.g., Chik et al., 2021)—demand advances in standard curve modeling at low concentrations. Such work can aid public health decision-making by helping to determine the sensitivity to detect a meaningful change in concentration on one hand or if an observed change can be explained by random variation alone on the other. This is particularly true in cases where, for whatever reason, digital PCR is not being used and in which explicitly quantifying the uncertainty of qPCR may help data users in their choice of appropriate methodology.

Building on conventional qPCR theory and foundational quantitative microbiology theory involving Poisson processes, this work mechanistically describes the non-linear pattern and increased variability of *Cq* values observed at low concentrations as well as non-detects. Critically, the developed model is not an empirical model that requires large amounts of data to describe phenomena with more numerous fitted parameters and that may have limited application outside intensely studied case-specific scenarios. The enhanced standard curve model is a mechanistic model developed from theoretical principles that should be no less broadly applicable than the MPN approach to estimating concentrations and adds no case-specific fitting parameters. This model describes why non-detects and changing patterns of *Cq* values occur at low concentrations, facilitates model fitting without arbitrary decisions about which data to exclude from linear modeling, and enables characterization of the uncertainty in fitted parameters for which only point estimates have typically been provided.

With continued efforts to validate and develop this type of modeling framework (including packaging tools to improve their accessibility to practitioners), this approach can unlock additional value in qPCR-based quantification outside of the linear dynamic range, whether it is applied to estimating gene concentrations in various water matrices or more generally quantifying gene abundances in other contexts. Moreover, it can aid quantification of the uncertainty in all qPCR-based results rather than merely reporting point-estimates mapped from a linear standard curve model. It provides a path forward to improve qPCR data quality by developing guidelines for standard curve experiment design and inter-lab comparison. It also provides a foundation from which to explore additional random errors in qPCR such as losses in the concentration/purification and extraction processes that may depend on matrix effects and a means to extract as much value as possible out of available data. These activities will improve the utility of qPCR to generate epidemiologically meaningful trends in the context of wastewater monitoring of SARS-CoV-2 and other pathogens and to quantify exposures in microbial risk assessments to advance the protection of public health. More generally, it will improve the quantitative value of qPCR beyond detecting order-of-magnitude relative differences.

## Data availability statement

The original contributions presented in this study are included in the article/Supplementary material, further inquiries can be directed to the corresponding author.

## Author contributions

PS, AC, and ME conceived the need for this work. ME and PS developed the framework for completing this work, secured the funding, and drafted the manuscript. PS conducted the analysis. NA, PD’A, RD, HD, MG, CH, JK, CM, X-LP, SP, NP, YQ, and MS contributed to the data and perspectives regarding laboratory practices. HD and MS conducted the supporting experimental analyses (described in the section “5. Application of model to analysis of experimental standard curve data”). All authors contributed to the article and approved the submitted version.
