# Supplementary material for: Realizing the value in “non-standard” parts of the qPCR standard curve by integrating fundamentals of quantitative microbiology
Source: Front Microbiol. 2023 Mar 3;14:1048661. doi: 10.3389/fmicb.2023.1048661 (PMC10020645; doi:10.3389/fmicb.2023.1048661)
Supplement: Supplementary file 1 [file Data_Sheet_1.docx]

Supplementary Content

# Derivation of Equations 8–10

Equation 4 describes a normal distribution of $Cq$ values given $Intercept$, $Slope$, an initial number of target genes ($N_{0}$) and $Cq$ residual standard deviation ($\sigma$).

$$Cq \sim N\left( Intercept+Slope\times\log_{10} N_{0},\sigma^{2} \right)$$

The corresponding probability density function is as follows:

$$f\left( Cq \right)=\frac{1}{\sqrt{2\pi}\sigma}\exp\left\{ -\frac{\left[ Cq-\left( Intercept+Slope\cdot\log_{10} N_{0} \right) \right]^{2}}{2\sigma^{2}} \right\}$$

Because $N_{0}$ is a random variable rather than a known value, the above equation is a conditional probability, $f\left( Cq|N_{0} \right)$. To determine the unconditional (or marginal) probability of $Cq$, the law of total probability may be applied. Because $\log_{10} N_{0}$ is undefined for $N_{0}=0$, only $N_{0}\geq1$ are considered for numerical $Cq$ values. Given a Poisson distribution for $N_{0}$ with concentration $\lambda$ in gene copies per reaction, the probability density of numerical $Cq$ values (equation 8) is:

$$f\left( Cq \right)=\sum_{N_{0}=1}^{\infty} f\left( Cq\cap N_{0} \right)=\sum_{N_{0}=1}^{\infty} p\left( N_{0} \right)\times f\left( Cq|N_{0} \right)$$

$$f\left( Cq \right)=\sum_{N_{0}=1}^{\infty} \frac{e^{-\lambda}\lambda^{N_{0}}}{N_{0}!}\times\frac{1}{\sqrt{2\pi}\sigma}\exp\left\{ -\frac{\left[ Cq-\left( Intercept+Slope\cdot\log_{10} N_{0} \right) \right]^{2}}{2\sigma^{2}} \right\}$$

Assuming that non-detects ($ND$) result only from reactions containing no target genes to amplify ($N_{0}=0$), the probability of a reaction yielding no $Cq$ value (equation 9) is:

$$P\left( Cq=ND \right)=P\left( N_{0}=0 \right)=e^{-\lambda}$$

Finally, the cumulative distribution function of numerical $Cq$ values (equation 10) can be defined and simplified as follows, including use of $\Phi\left( . \right)$ notation for the cumulative distribution function of the standard normal distribution. R scripts for computation are shown below (Section 2.2).

$$F\left( Cq \right)=\int_{-\infty}^{Cq} \sum_{N_{0}=1}^{\infty} \frac{e^{-\lambda}\lambda^{N_{0}}}{N_{0}!}\times\frac{1}{\sqrt{2\pi}\sigma}\exp\left\{ -\frac{\left[ Cq-\left( Intercept+Slope\cdot\log_{10} N_{0} \right) \right]^{2}}{2\sigma^{2}} \right\}dx$$

$$F\left( Cq \right)=\sum_{N_{0}=1}^{\infty} \frac{e^{-\lambda}\lambda^{N_{0}}}{N_{0}!}\times\int_{-\infty}^{Cq} \frac{1}{\sqrt{2\pi}\sigma}\exp\left\{ -\frac{\left[ Cq-\left( Intercept+Slope\cdot\log_{10} N_{0} \right) \right]^{2}}{2\sigma^{2}} \right\}dx$$

$$F\left( Cq \right)=\sum_{N_{0}=1}^{\infty} \frac{e^{-\lambda}\lambda^{N_{0}}}{N_{0}!}\times\Phi\left( \frac{Cq-\left( Intercept+Slope\cdot\log_{10} N_{0} \right)}{\sigma} \right)$$

# Sample R Scripts

## Simulation of qPCR Data

qPCRSim <- function(NumSim, GCRxn, CqInt, PCREff, CqErr, Poisson, AmpErr) {

### FUNCTION DETAILS

# This function returns a random Cq value given the following:

# NumSim Number of simulated data to produce

# GCRxn Gene copies per reaction (Poisson mean or integer)

# CqInt Expected Cq given an integer dose of 1

# PCREff PCR efficiency (<=100%)

# CqErr Cq residual standard deviation

# Poisson Toggle for inclusion of well random sampling error

# AmpErr Toggle for inclusion of amplification error

#

# Philip J. Schmidt, PhD, A.Stat.

# June 4, 2022

### ERROR TRAPPING

# Ensure NumSim is a positive integer

if(!is.numeric(NumSim) | as.integer(NumSim)!=NumSim | NumSim <= 0)

stop('NumSim must be a positive integer')

# Ensure GCRxn is a non-negative number

if(any(!is.numeric(GCRxn)) | any(GCRxn < 0))

stop('GCRxn must be a non-negative number')

# Ensure CqInt is a positive number with a practical upper bound of 50

if(!is.numeric(CqInt) | CqInt < 0 | CqInt > 50)

stop('CqInt must be a positive number < 50')

# Ensure PCREff is a positive number between zero and one

if(!is.numeric(PCREff) | PCREff < 0 | PCREff > 1)

stop('PCREff must be a positive number between zero and one')

# Ensure CqErr is a positive number with a practical upper bound of 2

if(!is.numeric(CqErr) | CqErr < 0 | CqErr > 2)

stop('CqErr must be a positive number < 2')

# Ensure CqInt, PCREff, CqErr, Poisson, and AmpErr are not vectors

if(any(length(CqInt) > 1 | length(PCREff) > 1 | length(CqErr) > 1 |

length(Poisson) > 1 | length(AmpErr) > 1)==TRUE)

stop('Only Cq and GCRxn may be vectors')

# Ensure Poisson is boolean

if(Poisson %in% c(0, 1, TRUE, FALSE)==FALSE)

stop('Poisson must be boolean')

# Ensure AmpErr is boolean

if(AmpErr %in% c(0, 1, TRUE, FALSE)==FALSE)

stop('AmpErr must be boolean')

# Ensure GCRxn is an integer if Poisson==FALSE

if(Poisson==FALSE & any(as.integer(GCRxn)!=GCRxn))

stop('GCRxn must be a non-negative integer if Poisson = FALSE')

# Warn user if length of GCRxn is not either 1 or NumSim

if(length(GCRxn)!=1 & length(GCRxn)!=NumSim)

warning('GCRxn length is not equal to NumSim')

### Simulate Cq values

# Convert input GCRxn to N0 (number of instances of target gene before PCR)

if(Poisson==TRUE) {

N0 <- rpois(NumSim, GCRxn)

} else {

N0 <- GCRxn

}

# Determine expected value of Cq

if (AmpErr==FALSE) {

MuCq <- (CqInt * log10(1 + PCREff) - log10(N0)) / log10(1 + PCREff)

} else {

# Propogate random amplification error for 10 cycles

N1 <- N0 + rbinom(NumSim, N0, PCREff)

N2 <- N1 + rbinom(NumSim, N1, PCREff)

N3 <- N2 + rbinom(NumSim, N2, PCREff)

N4 <- N3 + rbinom(NumSim, N3, PCREff)

N5 <- N4 + rbinom(NumSim, N4, PCREff)

N6 <- N5 + rbinom(NumSim, N5, PCREff)

N7 <- N6 + rbinom(NumSim, N6, PCREff)

N8 <- N7 + rbinom(NumSim, N7, PCREff)

N9 <- N8 + rbinom(NumSim, N8, PCREff)

N10 <- N9 + rbinom(NumSim, N9, PCREff)

MuCq <- 10 + (CqInt * log10(1 + PCREff) - log10(N10))/log10(1 + PCREff)

}

# Add Cq residual error

Cq <- rnorm(NumSim, MuCq, CqErr)

# Replace Inf values resulting from non-detects (N0 = 0) with NaN

Cq[!is.finite(Cq)] <- NaN

### Output simulated data

return(Cq)

}

### Sample simulation similar to Fig. 5a

# Simulate Data

NumSim <- 101

GCRxn <- 10^(0:100/20-2) # Logarithmically spaced values 0.01-1000 gc/rxn

CqInt <- 36

PCREff <- 0.9

CqErr <- 0.25

Cq <- qPCRSim(NumSim, GCRxn, CqInt, PCREff, CqErr, Poisson=TRUE,

AmpErr=TRUE)

# Plot simulated data

plot(GCRxn, Cq, log = "x", xlab = "gc/rxn", ylab = "Cq",

xlim = c(0.01, 1000), xaxt = "n", ylim = c(24, 38), yaxt = "n",

col = "blue", main = "Simulated data (CqInt=36, PCREff=0.9, CqErr=0.25)",

sub = "Non-detects not shown", col.sub = "red")

axis(1, c(0.01, 0.1, 1, 10, 100, 1000))

axis(2, seq(from = 24, to = 38, by = 2), las = 1)

## Probability Calculations for qPCR Data

qPCRProb <- function(Cq, GCRxn, CqInt, PCREff, CqErr, CumProb) {

### FUNCTION DETAILS

# This function returns the probability density or cumulative density

# of a Cq value given:

# Cq Quantification cycle (or NaN for non-detect)

# GCRxn Concentration in gene copies per reaction

# CqInt Expected Cq given an integer dose of 1

# PCREff PCR efficiency (<=100%)

# CqErr Cq residual standard deviation

# CumProb Toggle for computation of cumulative density

#

# Philip J. Schmidt, PhD, A.Stat.

# June 4, 2022

### ERROR TRAPPING

# Ensure each Cq is a number between 0 and 50 or NaN for non-detects

if(any(!is.numeric(Cq)) | any(Cq[!is.nan(Cq)] <= 0)|

any(Cq[!is.nan(Cq)] > 50))

stop('Cq must be a number between 0 and 50 or NaN for non-detects')

# Ensure GCRxn is a non-negative number

if(any(!is.numeric(GCRxn)) | any(GCRxn < 0))

stop('GCRxn must be a non-negative number')

# Ensure CqInt is a positive integer with an practical upper bound of 50

if(!is.numeric(CqInt) | CqInt < 0 | CqInt > 50)

stop('CqInt must be a positive number < 50')

# Ensure PCREff is a positive number between zero and one

if(!is.numeric(PCREff) | PCREff < 0 | PCREff > 1)

stop('PCREff must be a positive number between zero and one')

# Ensure CqErr is a positive number with a practical upper bound of 2

if(!is.numeric(CqErr) | CqErr < 0 | CqErr > 2)

stop('CqErr must be a positive number < 2')

# Ensure CqInt, PCREff, CqErr, and CumProb are not vectors

if(any(length(CqInt) > 1 | length(PCREff) > 1 | length(CqErr) > 1 |

length(CumProb) > 1)==TRUE)

stop('Only Cq and GCRxn may be vectors')

# Ensure CumProb is boolean

if(CumProb %in% c(1, 2, TRUE, FALSE)==FALSE)

stop('CumProb must be TRUE (probability density) or FALSE (cumulative

density)')

# If GCRxn is not a single value, make sure it has same length as Cq

if(length(GCRxn) > 1 & length(GCRxn) != length(Cq))

stop('If GCRxn is a vector, it must have same length as Cq')

### Compute Probabilities

# Convert PCR efficiency to slope

Slope <- -1 / log10(1 + PCREff)

# Convert single GCRxn value to vector

if(length(GCRxn)==1) GCRxn <- rep(GCRxn, length(Cq))

# Arrange results in data frame & determine bounds of numerical integration

NumCq <- length(Cq)

DF <- data.frame(Cq, GCRxn)

DF$N0min <- qpois(rep(1E-15, NumCq), GCRxn)

DF$N0max <- qpois(rep(1-1E-15, NumCq), GCRxn)

# Bounds are widened to better encompass terms that are unlikely by

# Poisson but comparatively likely in the normal part of the integration

DF$N0min <- pmax(DF$N0min - round(DF$GCRxn - DF$N0min, 0), rep(1, NumCq))

DF$N0max <- DF$N0max + round(DF$N0max - DF$GCRxn, 0)

# Bounds are changed to zero for non-detects

DF$N0min[is.nan(DF$Cq)] <- 0

DF$N0max[is.nan(DF$Cq)] <- 0

# Function for term calculation

FUN <- function(N0, Cq, GCRxn, CqInt, Slope, CqErr, CumProb) {

if (is.nan(Cq)) {

dpois(0, GCRxn)

} else {

if (CumProb==TRUE) {

dpois(N0, GCRxn) * pnorm(Cq, CqInt + Slope * log10(N0), CqErr)

} else {

dpois(N0, GCRxn) * dnorm(Cq, CqInt + Slope * log10(N0), CqErr)

}

}

}

# Compute vector of consequential terms using loop (in case GCRxn varies)

for (i in 1:NumCq) {

N0 <- seq(from = DF$N0min[i], to = DF$N0max[i])

terms <- FUN(N0, DF$Cq[i], DF$GCRxn[i], CqInt, Slope, CqErr, CumProb)

sum <- sum(terms)

if (CumProb==TRUE) {

DF$CumProb[i] <- sum

} else {

DF$ProbDens[i] <- sum

}

# Compute truncation measure (maximum value of outermost terms summed)

if (is.nan(DF$Cq[i])) {DF$Trunc[i] <- 0}

else {

if(DF$N0min[i] > 1) {

DF$Trunc[i] <- max(terms[1] / sum, terms[length(terms)] / sum)

} else {

DF$Trunc[i] <- terms[length(terms)] / sum

}

}

}

return(DF)

}

### Sample Calculations

# Compute PDF & CDF of Cq values (excluding calculation of non-detect prob.)

Cq <- seq(from = 30, to = 38, by = 0.01)

GCRxn <- 2; CqInt <- 36; PCREff <- 0.9; CqErr <- 0.25

PDF <- qPCRProb(Cq, GCRxn, CqInt, PCREff, CqErr, CumProb = 0)

CDF <- qPCRProb(Cq, GCRxn, CqInt, PCREff, CqErr, CumProb = 1)

# Plot PDF and CDF

plot(PDF$Cq, PDF$ProbDens, type = "l", xlab = "Cq",

ylab = "Probability Density", xlim = c(30, 38), col = "blue",

main = "PDF (GCRxn = 2, CqInt = 36, PCREff = 0.9, CqErr = 0.25)",

sub = "Does not integrate to 1 due to non-detects", col.sub = "red")

plot(CDF$Cq, CDF$CumProb, type = "l", xlab = "Cq", ylab =

"Cumulative Density", xlim = c(30, 38), col = "blue", ylim = c(0, 1),

main = "CDF (GCRxn = 2, CqInt = 36, PCREff = 0.9, CqErr = 0.25)",

sub = "Plateaus below 1 due to non-detects", col.sub = "red")

## Maximum Likelihood Fitting of Standard Curve Model

MLE.SC <- function(Cq, GCRxn, CqInt.Guess, PCREff.Guess, CqErr.Guess) {

### FUNCTION DETAILS

# This function returns MLEs of the standard curve parameters given:

# Cq Quantification cycle (or NaN for non-detect)

# GCRxn Concentration in gene copies per reaction

# CqInt.Guess Initial value of expected Cq given an integer dose of 1

# PCREff.Guess Initial value of PCR efficiency (<=100%)

# CqErr.Guess Initial value of Cq residual standard deviation

#

# Philip J. Schmidt, PhD, A.Stat.

# June 8, 2022

### ERROR TRAPPING

# Ensure each Cq is a number between 0 and 50 or NaN for non-detects

if(any(!is.numeric(Cq)) | any(Cq[!is.nan(Cq)] <= 0)|

any(Cq[!is.nan(Cq)] > 50))

stop('Cq must be a number between 0 and 50 or NaN for non-detects')

# Ensure GCRxn is a non-negative number

if(any(!is.numeric(GCRxn)) | any(GCRxn < 0))

stop('GCRxn must be a non-negative number')

# Ensure GCRxn and Cq are vectors of the same length

if(length(GCRxn) != length(Cq))

stop('GCRxn must have same length as Cq')

# Ensure CqInt.Guess is positive integer with a practical upper bound of 50

if(!is.numeric(CqInt.Guess) | CqInt.Guess < 0 | CqInt.Guess > 50)

stop('CqInt.Guess must be a positive number < 50')

# Ensure PCREff.Guess is a positive number between zero and one

if(!is.numeric(PCREff.Guess) | PCREff.Guess < 0 | PCREff.Guess > 1)

stop('PCREff.Guess must be a positive number between zero and one')

# Ensure CqErr.Guess is a positive number with a practical upper bound of 2

if(!is.numeric(CqErr.Guess) | CqErr.Guess < 0 | CqErr.Guess > 2)

stop('CqErr.Guess must be a positive number < 2')

# Ensure CqInt, PCREff, and CqErr are not vectors

if(any(length(CqInt.Guess) > 1 | length(PCREff.Guess) > 1 |

length(CqErr.Guess) > 1)==TRUE)

stop('Only Cq and GCRxn may be vectors')

### TRANSFORM INITIAL VALUES

# Values are non-negative with upper bounds 50, 1, 2 (see error trapping)

inits <- qnorm(c(CqInt.Guess/50, PCREff.Guess, CqErr.Guess/2))

### COMPUTE NEGATIVE LOG-LIKELIHOOD FOR MINIMIZATION

lnL <- function(data, par) {

CqInt <- pnorm(par[1]) * 50

PCREff <- pnorm(par[2])

CqErr <- pnorm(par[3]) * 2

DF <- qPCRProb(data, GCRxn, CqInt, PCREff, CqErr, FALSE)

DF$lnL <- log(DF$ProbDens)

return(-sum(DF$lnL))

}

### IMPLEMENT MINIMIZATION AND DISPLAY RESULTS

opt <- optim(par = inits, fn = lnL, data = Cq)

mle <- c(50, 1, 2) * pnorm(opt$par, 0, 1)

CqInt <- mle[1]; PCREff <- mle[2]; CqErr <- mle[3]; lnL <- -opt$value

MLEfit <- setNames(c(CqInt, PCREff, CqErr, lnL),

c("CqInt", "PCREff", "CqErr", "lnL"))

print(MLEfit)

return(MLEfit)

}

### Compute MLEs of Figure 6 data (results differ slightly due to rounded data)

# Load data (rounded to two decimal places)

Cq <- c(30.29, 29.96, 29.91, 29.88, 30.09, 30.18, 29.79, 29.94, 30.10,

30.97, 31.00, 30.94, 31.22, 30.54, 30.83, 30.76, 30.91, 30.87,

32.45, 32.22, 32.09, 32.05, 32.06, 31.87, 32.09, 31.86, 31.73,

32.70, 33.14, 32.90, 33.19, 32.52, 33.01, 32.72, 33.08, 32.79,

34.92, 34.09, 33.65, 34.18, 34.35, 33.25, 33.97, 33.94, 34.18,

34.77, 34.86, 36.09, 35.55, 35.10, 34.85, 35.59, 34.43, 35.07,

35.71, NaN, 36.23, 39.11, 36.46, 35.70, 36.63, 36.28, 36.20,

37.67, 37.65, NaN, 37.03, 35.44, 37.18, 36.76, 38.09, 37.20,

38.10, NaN, 36.95, 37.87, 38.02, NaN, NaN, 40.46, 37.99,

38.82, NaN, 39.06, NaN, NaN, NaN, NaN, NaN, NaN)

GCRxn <- c(rep(200, 9), rep(100, 9), rep(50, 9), rep(25, 9), rep (12.5, 9),

rep(6.25, 9), rep(3.125, 9), rep(1.5625, 9), rep(0.78125, 9),

rep(0.390625, 9))

# Plot data

plot(GCRxn, Cq, log = "x", xlab = "gc/rxn", ylab = "Cq",

xlim = c(0.1, 1000), xaxt = "n", ylim = c(28, 42), yaxt = "n",

col = "blue", main = "Figure 6 Data", sub = "Non-detects not shown",

col.sub = "red")

axis(1, c(0.01, 0.1, 1, 10, 100, 1000))

axis(2, seq(from = 28, to = 42, by = 2), las = 1)

# MLE

MLE.SC(Cq, GCRxn, 36, 0.9, 0.25)

# Sample OpenBUGS Script to Assess Standard Curve Model Parametric Uncertainty

### PARAMETER DEFINITIONS - Standard curve analysis

logNq: logarithm of number of amplicons determining Cq - STOCHASTIC VARIABLE

Nq: signal strength determining Cq - DETERMINISTIC VARIABLE OF INTEREST

E: PCR efficiency - STOCHASTIC VARIABLE OF INTEREST

log2sigma: base-2 log of residual Cq standard deviation – STOCHASTIC VARIABLE

sigma: minimal standard deviation of normal error in Cq about expected value

- DETERMINISTIC VARIABLE OF INTEREST

intercept: intercept of log-linear model - DETERMINISTIC

slope: slope of log-linear model - DETERMINISTIC

Y[i]: #gc in well i subjected to PCR - STOCHASTIC NUISANCE VARIABLE

lambda[i]: expected #gc in well i - CONTROL VARIABLE

ND[i]: indicator variable for non-detect in well i - MEASURED VARIABLE

mu[i]: mean of Cq distribution for well i - DETERMINISTIC

tau[i]: inverse variance of error in Cq - DETERMINISTIC

Cq[i]: Cq value of well i - MEASURED STOCHASTIC VARIABLE (non-detect Cq = -1)

model {

### Standard curves analysis

# Define priors

logNq ~ dunif(0, 15) # 2^50 is approximately 1E15

Nq <- pow(10, logNq)

E ~ dunif(0, 1) # Coerces PCR efficiency <1

log2sigma ~ dunif(-3, 1) # sigma < 2 Cq

sigma <- pow(2, log2sigma)

intercept <- log(Nq) / log(1 + E)

slope <- -log(10) / log(1 + E)

# Define likelihood

for (i in 1:m) {

# Work-around to coerce Y=0 for non-detects and Y>0 otherwise

Y_branch[i, 1] ~ dpois(lambda[i]) T(1,)

Y_branch[i, 2] ~ dpois(lambda[i]) T(0, 0)

Y[i] <- Y_branch[i, ND[i] + 1]

# Work-around to avoid log(0) error when Y=0

mu_branch[i, 1] <- (log(Nq) - log(Y[i])) / log(1 + E)

mu_branch[i, 2] <- -1 # Assign impossible Cq when Y=0

mu[i] <- mu_branch[i, ND[i] + 1]

# Work-around to dissociate non-detects from estimation of tau

tau_branch[i, 1] <- pow(sigma, -2)

tau_branch[i, 2] <- 1000000

tau[i] <- tau_branch[i, ND[i] + 1]

Cq[i] ~ dnorm(mu[i], tau[i]) # Small impossible Cq for non-detects

}

}

EXAMPLE DATA

list(m = 4)

lambda[] Cq[] ND[]

8 34 0

4 35 0

2 36 0

1 -1 1

END

EXAMPLE INITS

list(logdelta = 12, phi = 1, log2sigma = 0)

# Supplementary Data

Table S1: Simulated standard curve data ($n=101$) with (Fig 5a) and without (Fig 5b) random amplification error (RAE). Data were simulated with intercept 36, $E=0.9$, and $\sigma=0.25$.
N_0_ = initial integer number of target genes and “ND” = non-detect.

| log gc/rxn | N_0_ | Cq Error | Cq No RAE | Cq With RAE |  | log gc/rxn | N_0_ | Cq Error | Cq No RAE | Cq With RAE |
| --- | --- | --- | --- | --- | --- | --- | --- | --- | --- | --- |
| -2.00 | 0 | N/A | ND | ND |  | 0.55 | 0 | N/A | ND | ND |
| -1.95 | 0 | N/A | ND | ND |  | 0.60 | 5 | -0.0014 | 33.4911 | 33.5692 |
| -1.90 | 0 | N/A | ND | ND |  | 0.65 | 6 | -0.1948 | 33.0137 | 33.2265 |
| -1.85 | 0 | N/A | ND | ND |  | 0.70 | 3 | 0.4331 | 34.7215 | 34.8736 |
| -1.80 | 0 | N/A | ND | ND |  | 0.75 | 5 | -0.3463 | 33.1462 | 33.1200 |
| -1.75 | 0 | N/A | ND | ND |  | 0.80 | 8 | 0.1400 | 32.9003 | 33.0170 |
| -1.70 | 0 | N/A | ND | ND |  | 0.85 | 10 | -0.0106 | 32.4020 | 32.3309 |
| -1.65 | 0 | N/A | ND | ND |  | 0.90 | 4 | -0.1844 | 33.6557 | 33.8445 |
| -1.60 | 0 | N/A | ND | ND |  | 0.95 | 12 | -0.0097 | 32.1188 | 32.0001 |
| -1.55 | 0 | N/A | ND | ND |  | 1.00 | 8 | 0.1885 | 32.9488 | 32.7580 |
| -1.50 | 0 | N/A | ND | ND |  | 1.05 | 9 | -0.1219 | 32.4549 | 32.4932 |
| -1.45 | 0 | N/A | ND | ND |  | 1.10 | 7 | 0.0215 | 32.9898 | 33.1047 |
| -1.40 | 0 | N/A | ND | ND |  | 1.15 | 15 | -0.5308 | 31.2501 | 31.3781 |
| -1.35 | 0 | N/A | ND | ND |  | 1.20 | 12 | -0.5415 | 31.5871 | 31.5947 |
| -1.30 | 0 | N/A | ND | ND |  | 1.25 | 22 | -0.1498 | 31.0344 | 31.0426 |
| -1.25 | 0 | N/A | ND | ND |  | 1.30 | 27 | -0.0124 | 30.8528 | 30.9078 |
| -1.20 | 0 | N/A | ND | ND |  | 1.35 | 25 | 0.0512 | 31.0362 | 31.0592 |
| -1.15 | 0 | N/A | ND | ND |  | 1.40 | 23 | 0.2099 | 31.3249 | 31.3859 |
| -1.10 | 0 | N/A | ND | ND |  | 1.45 | 31 | 0.2702 | 30.9201 | 30.8578 |
| -1.05 | 0 | N/A | ND | ND |  | 1.50 | 31 | 0.4153 | 31.0652 | 31.0623 |
| -1.00 | 0 | N/A | ND | ND |  | 1.55 | 42 | -0.3014 | 29.8753 | 29.8194 |
| -0.95 | 0 | N/A | ND | ND |  | 1.60 | 55 | -0.0726 | 29.6840 | 29.7090 |
| -0.90 | 1 | 0.1755 | 36.1755 | 35.8602 |  | 1.65 | 29 | -0.5260 | 30.2278 | 30.4137 |
| -0.85 | 0 | N/A | ND | ND |  | 1.70 | 47 | -0.3449 | 29.6566 | 29.5989 |
| -0.80 | 1 | 0.1634 | 36.1634 | 35.8092 |  | 1.75 | 54 | -0.0011 | 29.7841 | 29.7712 |
| -0.75 | 0 | N/A | ND | ND |  | 1.80 | 52 | -0.1040 | 29.7400 | 29.7926 |
| -0.70 | 0 | N/A | ND | ND |  | 1.85 | 79 | 0.3488 | 29.5413 | 29.5147 |
| -0.65 | 0 | N/A | ND | ND |  | 1.90 | 82 | 0.3743 | 29.5087 | 29.5546 |
| -0.60 | 0 | N/A | ND | ND |  | 1.95 | 94 | 0.1760 | 29.0976 | 29.0695 |
| -0.55 | 0 | N/A | ND | ND |  | 2.00 | 104 | 0.2782 | 29.0423 | 29.0791 |
| -0.50 | 0 | N/A | ND | ND |  | 2.05 | 99 | 0.2815 | 29.1224 | 29.1218 |
| -0.45 | 0 | N/A | ND | ND |  | 2.10 | 130 | 0.0576 | 28.4741 | 28.4307 |
| -0.40 | 1 | -0.3326 | 35.6674 | 35.5049 |  | 2.15 | 143 | 0.2351 | 28.5031 | 28.5021 |
| -0.35 | 0 | N/A | ND | ND |  | 2.20 | 164 | 0.0386 | 28.0931 | 28.0840 |
| -0.30 | 1 | 0.0488 | 36.0488 | 35.8219 |  | 2.25 | 171 | 0.0180 | 28.0074 | 28.0578 |
| -0.25 | 2 | -0.0162 | 34.9039 | 35.1534 |  | 2.30 | 206 | 0.3565 | 28.0557 | 28.0512 |
| -0.20 | 1 | -0.1973 | 35.8027 | 35.9755 |  | 2.35 | 236 | -0.0674 | 27.4200 | 27.4491 |
| -0.15 | 1 | -0.2125 | 35.7875 | 35.5630 |  | 2.40 | 254 | 0.1473 | 27.5202 | 27.5105 |
| -0.10 | 0 | N/A | ND | ND |  | 2.45 | 299 | 0.2303 | 27.3491 | 27.3830 |
| -0.05 | 0 | N/A | ND | ND |  | 2.50 | 307 | 0.4924 | 27.5700 | 27.5636 |
| 0.00 | 2 | 0.4261 | 35.3462 | 35.0651 |  | 2.55 | 353 | 0.1014 | 26.9615 | 26.9562 |
| 0.05 | 1 | -0.2326 | 35.7674 | 35.5340 |  | 2.60 | 402 | -0.1287 | 26.5289 | 26.5394 |
| 0.10 | 1 | 0.4382 | 36.4382 | 36.3365 |  | 2.65 | 423 | -0.1860 | 26.3923 | 26.4061 |
| 0.15 | 1 | -0.1833 | 35.8167 | 35.8594 |  | 2.70 | 483 | -0.0952 | 26.2764 | 26.2641 |
| 0.20 | 1 | 0.1338 | 36.1338 | 36.7740 |  | 2.75 | 530 | -0.4494 | 25.7776 | 25.7979 |
| 0.25 | 3 | 0.1929 | 34.4813 | 34.2842 |  | 2.80 | 674 | 0.5687 | 26.4212 | 26.4180 |
| 0.30 | 5 | 0.3081 | 33.8007 | 33.8225 |  | 2.85 | 682 | -0.3254 | 25.5087 | 25.5072 |
| 0.35 | 2 | 0.3662 | 35.2863 | 35.2291 |  | 2.90 | 788 | 0.2531 | 25.8621 | 25.8598 |
| 0.40 | 4 | -0.5599 | 33.2802 | 33.1403 |  | 2.95 | 874 | -0.1185 | 25.3291 | 25.3087 |
| 0.45 | 1 | -0.1380 | 35.8620 | 35.5969 |  | 3.00 | 994 | 0.2964 | 25.5436 | 25.5423 |
| 0.50 | 2 | 0.1968 | 35.1169 | 35.3019 |  |  |  |  |  |  |

Table S2: Experimental standard curve data ($n=90$) shown in Figures 7 and 8. Six no template controls resulting in no amplification are not shown. “ND” = non-detect.

| N1 copies/rxn | Cq values (9 technical replicates) | | | | | | | | |
| --- | --- | --- | --- | --- | --- | --- | --- | --- | --- |
| 200 | 30.29 | 29.96 | 29.91 | 29.88 | 30.09 | 30.18 | 29.79 | 29.94 | 30.10 |
| 100 | 30.97 | 31.00 | 30.94 | 31.22 | 30.54 | 30.83 | 30.76 | 30.91 | 30.87 |
| 50 | 32.45 | 32.22 | 32.09 | 32.05 | 32.06 | 31.87 | 32.09 | 31.86 | 31.73 |
| 25 | 32.70 | 33.14 | 32.90 | 33.19 | 32.52 | 33.01 | 32.72 | 33.08 | 32.79 |
| 12.5 | 34.92 | 34.09 | 33.65 | 34.18 | 34.35 | 33.25 | 33.97 | 33.94 | 34.18 |
| 6.25 | 34.77 | 34.86 | 36.09 | 35.55 | 35.10 | 34.85 | 35.59 | 34.43 | 35.07 |
| 3.125 | 35.71 | ND | 36.23 | 39.11 | 36.46 | 35.70 | 36.63 | 36.28 | 36.20 |
| 1.5625 | 37.67 | 37.65 | ND | 37.03 | 35.44 | 37.18 | 36.76 | 38.09 | 37.20 |
| 0.7813 | 38.10 | ND | 36.95 | 37.87 | 38.02 | ND | ND | 40.46 | 37.99 |
| 0.3906 | 38.82 | ND | 39.06 | ND | ND | ND | ND | ND | ND |

Table S3: qPCR conditions used for experimental data summarized in Section 5

|  | Time | Temp (^o^C) | Cycles |
| --- | --- | --- | --- |
| UNG Incubation | 2 min | 25 | 1x |
| Reverse Transcription | 15 min | 50 | 1x |
| Polymerase Activation | 2 min | 95 | 1x |
| Denature | 3 sec | 95 | 45x |
| Amplification | 30 sec | 55 |  |
